# Removal of the mechanoprotective influence of the cytoskeleton reveals PIEZO1 is gated by bilayer tension

**DOI:** 10.1038/ncomms10366

**Published:** 2016-01-20

**Authors:** Charles D. Cox, Chilman Bae, Lynn Ziegler, Silas Hartley, Vesna Nikolova-Krstevski, Paul R. Rohde, Chai-Ann Ng, Frederick Sachs, Philip A. Gottlieb, Boris Martinac

**Affiliations:** 1 Victor Chang Cardiac Research Institute, Darlinghurst, New South Wales 2010, Australia; 2 Department of Physiology and Biophysics, State University of New York at Buffalo, Buffalo, New York 14214, USA; 3 St Vincent's Clinical School, University of New South Wales, Darlinghurst, New South Wales 2010, Australia; 4 The Centre for Single Molecule Biophysics, State University of New York at Buffalo, Buffalo, New York 14214, USA

## Abstract

Mechanosensitive ion channels are force-transducing enzymes that couple mechanical stimuli to ion flux. Understanding the gating mechanism of mechanosensitive channels is challenging because the stimulus seen by the channel reflects forces shared between the membrane, cytoskeleton and extracellular matrix. Here we examine whether the mechanosensitive channel PIEZO1 is activated by force-transmission through the bilayer. To achieve this, we generate HEK293 cell membrane blebs largely free of cytoskeleton. Using the bacterial channel MscL, we calibrate the bilayer tension demonstrating that activation of MscL in blebs is identical to that in reconstituted bilayers. Utilizing a novel PIEZO1–GFP fusion, we then show PIEZO1 is activated by bilayer tension in bleb membranes, gating at lower pressures indicative of removal of the cortical cytoskeleton and the mechanoprotection it provides. Thus, PIEZO1 channels must sense force directly transmitted through the bilayer.

## Abstract (web summary)

PIEZO1 is a mechanosensitive ion channel, but the mechanism of force transduction is unknown. Here Cox and Bae *et al.* disrupt the cortical cytoskeleton in HEK293 cells to show that PIEZO1 is gated directly by membrane tension.

## Graphical abstract

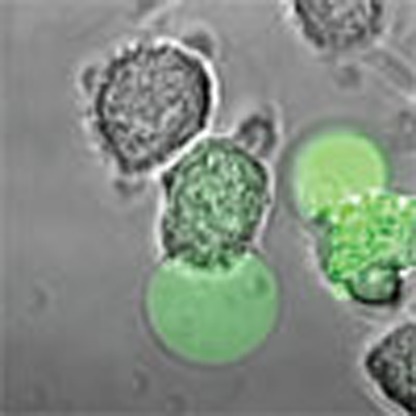

PIEZO channels are a recently cloned class of eukaryotic mechanosensitive channels identified initially in a murine neuroblastoma cell line (neuro-2a cells)^1^,^2^. The two subtypes, PIEZO1 and PIEZO2, are currently the subject of intense research and have been shown to be involved in numerous physiological and pathophysiological processes. PIEZO2 has been linked to light touch via expression in Merkel cells in the skin^3^,^4^. PIEZO1 has been linked to the maintenance of cell volume in erythrocytes and its dysfunction has been linked to specific hereditary anemias (for example, xerocytosis)^5^,^6^,^7^. In addition, it plays a role in vascular development, vascular physiology and cell differentiation^8^,^9^,^10^.

Identifying force transduction mechanisms in mechanosensitive channels has been difficult owing to the complex environment of a cell membrane patch and partly owing to the multiple pathways leading to mechanosensitive channel activation^11^,^12^,^13^,^14^,^15^. For example, mechanosensitive channels might be gated (i) directly by bilayer tension as seen with the MscS and MscL bacterial channel families as well as the two-pore domain K^+^ channels TREK-1 and TRAAK^16^,^17^ (This is referred to as the ‘Force-from-lipids' concept^18^,^19^), or (ii) by tethering to the cytoskeleton and/or the extracellular matrix^20^,^21^,^22^. It is important to note that even if a channel is gated directly by bilayer tension, the tension in the bilayer can be modified by cytoskeletal proteins and linkages to the extracellular matrix. Thus the sensitivity of a channel to the mean stress in the cortex can be modified by alterations in an array of scaffold proteins^23^. One way to probe the role of the cytoskeleton in the activation of a mechanosensitive channel is to use cytoskeletal deficient membrane ‘blebs'.

Blebs are rounded protrusions of the bilayer where connections between the cytoskeleton and the bilayer are largely broken^24^,^25^,^26^. They are found in numerous processes including cytokinesis, cell motility and programmed cell death^25^,^27^,^28^. The blebbing process itself is dynamic and tightly regulated by numerous factors. In a physiological setting, the lifetime of a bleb ranges from tens of seconds to minutes^25^,^26^. Their formation may be induced by numerous stimuli including changes in external osmolarity. Bleb formation usually starts with a breakdown of cortical f-actin (Fig. 1a)^25^,^29^. Subsequent bleb growth is modulated by hydrostatic pressure and actomyosin contractility. During the expansion phase, the bleb membrane is still amenable to electrophysiological study^30^,^31^,^32^,^33^. The challenge for electrophysiology is to maintain the decoupling of the cortical cytoskeleton long enough to measure channel activity since the cytoskeleton can be rebuilt^26^. Using this research paradigm, we asked whether the PIEZO1 channel is gated by bilayer tension.

We first generated blebs deficient in f-actin and β-tubulin long enough for electrophysiological recording. We then characterized the mechanical environment of the blebbed membranes using as a probe MscL, a mechanosensitive channel gated directly by bilayer tension^34^. The pipette suction required to gate MscL in blebs was three times less than in cell-attached patches, reflecting the fact that much of the cortical tension is supported by the cytoskeleton^23^. Interestingly, the gating of MscL in blebs was almost identical to that observed in liposomes. To apply the same experimental paradigm to PIEZO1 channels, we created a green fluorescent protein (GFP) fusion construct of PIEZO1 (with the fluorophore inserted in the centre of the protein at position 1591), and showed that not only do these channels behave like wild-type (WT) PIEZO1 channels but they are present in the membrane blebs and can be activated by applied negative pressure to the patch pipette. Like MscL, PIEZO1 channels in blebs were more sensitive to applied tension than in cell-attached patches. Thus PIEZO1 appears to sense tension in the bilayer and is gated according to the “force-from lipids” principle, an evolutionarily conserved gating mechanism.

## Results

### Blebbing HEK293 cells

**Figure 1:**
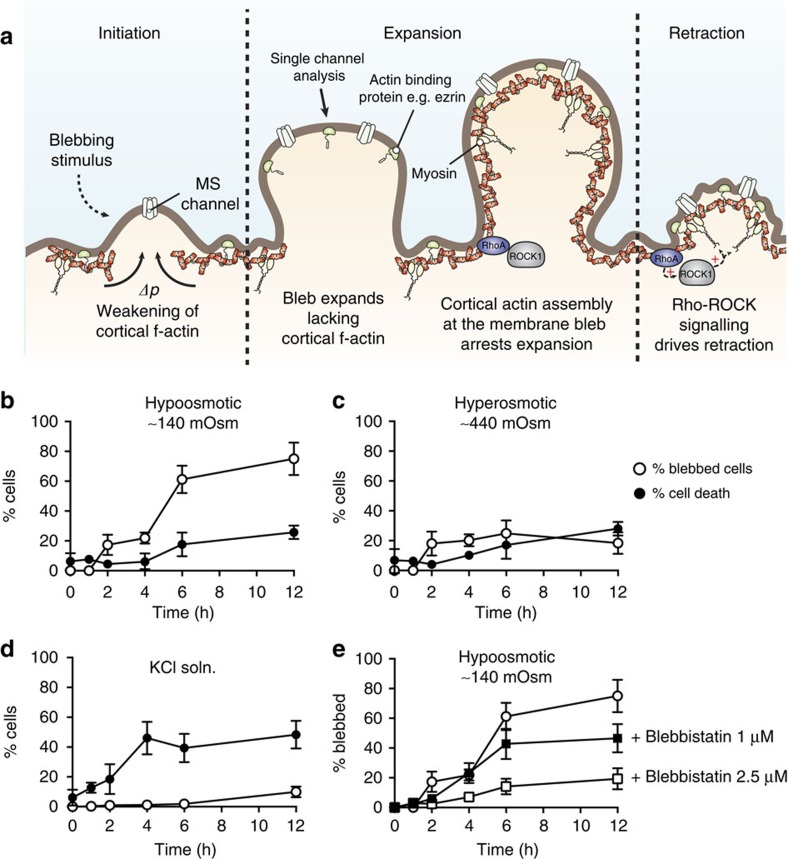
Overview of membrane bleb formation and the efficiency of blebbing solutions on HEK293 cells including the effect on cell viability. (**a**) Formation of membrane blebs consists of three phases: initiation, expansion and retraction. Initiation of blebs can be instigated by a variety of stimuli, and certain cell lines continually bleb (for example, M12 cell line^68^). The initiation phase usually involves a focal weakening or rupture of the cortical cytoskeleton and bleb expansion continues largely devoid of cortical f-actin driven by hydrostatic pressure^69^ and actomyosin contractility. Once polymerization of f-actin begins at the bleb membrane, expansion is halted. In the physiological setting, Rho-ROCK signalling then drives bleb retraction again via actomyosin contractility. Illustration of the number of cells blebbing and the corresponding % cells stained with trypan blue in response to treatment with (**b**) a hypoosmotic NaGluconate solution (∼140 mOsm), (**c**) a hyperosmotic NaGluconate solution (∼440 mOsm) and (**d**) a Ca^2+^ free KCl-based solution (first described for use in myocytes^35^,^70^). (**e**) Illustration of the dependence of blebbing on the activity of myosin II with almost complete abolition of bleb formation in the presence of 2.5 μM blebbistatin (data points represent mean±s.e.m.; *n*=4 with each replicate including >250 cells).

We first explored the best way to make stable blebs^30^,^31^,^33^. We focused on three solutions: (1) Hypoosmotic sodium gluconate solution (∼140 mOsm), (2) Hyperosmotic sodium gluconate (∼440 mOsm), and (3) KCl Ca^2+^ free solution^35^. The results were summarized by the number of cells that blebbed at various time points up to 12 h (Fig. 1b–e). The membrane integrity of cells after blebbing was monitored using a trypan blue assay. While the KCl Ca^2+^ free solution generated blebs, a high percentage of cells (>50%) stained with trypan blue after ∼4 h. The most effective solution was hypoosmotic sodium gluconate with >60% cells exhibiting at least one bleb after 6 h. Furthermore, this blebbing seemed to be dependent on the activity of myosin II based on the fact that blebbistatin greatly reduced the number of blebs (Fig. 1e).

### Cytoskeletal deficiency in HEK293 membrane blebs

**Figure 2:**
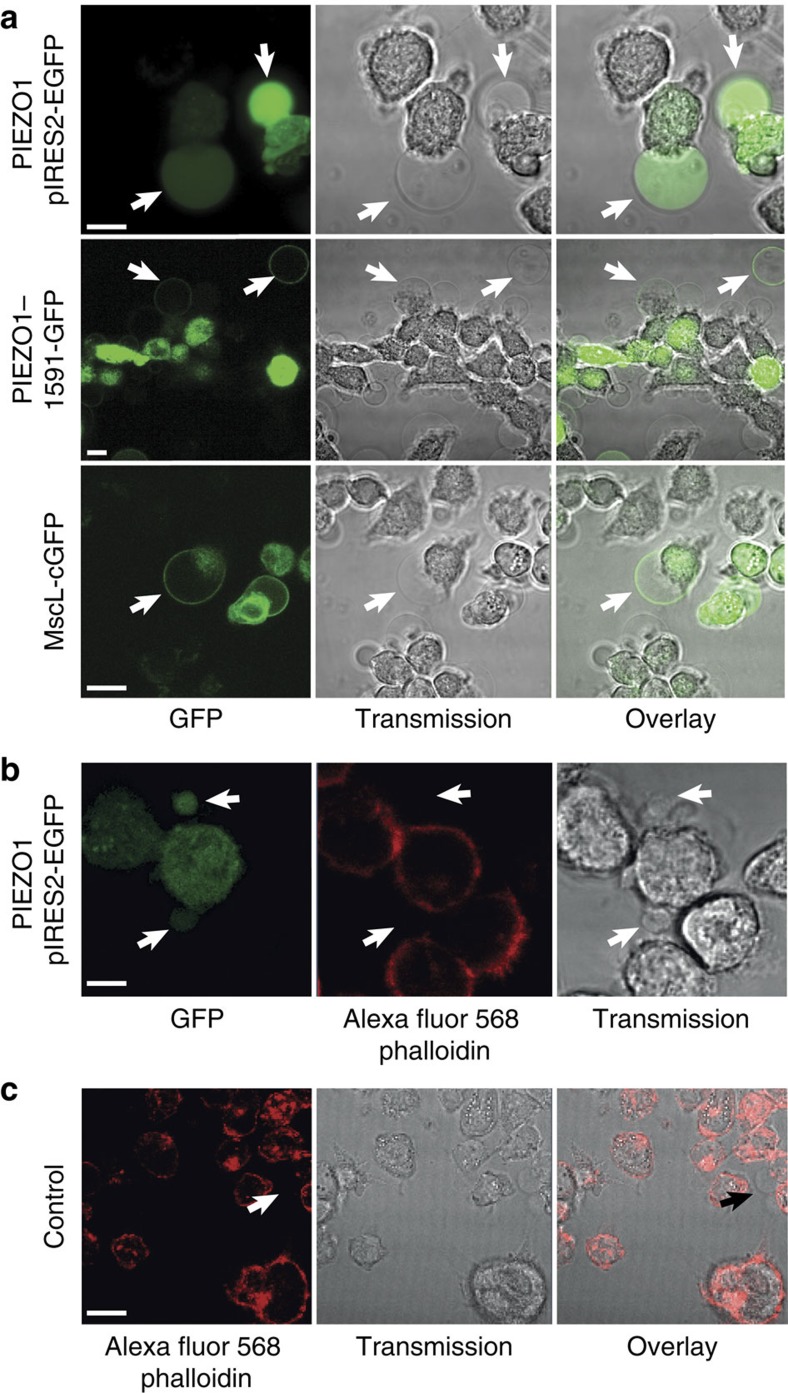
Fluorescence imaging of HEK293 cell membrane blebs generated using a hypo-osmotic solution. (**a**) Example of pIRES2–EGFP PIEZO1 expression in HEK blebs. The blebs can be clearly seen with free GFP inside the cytoplasm. Blebs sometimes grew to diameters greater than 15 μM (upper row). GFP fusion proteins of PIEZO1 (middle row) and MscL (lower row) can also be seen in the bleb membrane (scale bar, 10 μm). (**b**) Illustration of staining of cells expressing a pIRES2–EGFP PIEZO1 construct. Arrows represent blebs that contain GFP but are devoid of f-actin staining (red; scale bar, 7 μm). (**c**) As a control, we also stained cells blebbed in the same manner and stained with phalloidin. Again, the arrow represents a bleb that is not stained and hence deficient in f-actin (scale bar, 20 μm).

Next we investigated whether the resulting blebs were deficient in cortical cytoskeleton. We assayed f-actin using an Alexa Fluor 568 phalloidin conjugate. We also visualized three GFP containing constructs expressed in HEK293 cells: a pIRES2–EGFP PIEZO1 where GFP is expressed separately from the PIEZO1 channel, and two fusion proteins, one of PIEZO1 and one of MscL (Fig. 2a and Supplementary Fig. 1). All the images show large rounded protrusions that grew up to 15 μm in diameter. It was simple to identify the GFP fluorescence of the fusion proteins at the membrane boundary. Figure 2b shows blebs from cells expressing pIRES2–EGFP PIEZO1 where free GFP was present in the cytoplasm and the cell was also stained with phalloidin. The arrows show blebs with GFP fluorescence but f-actin is not visible (Fig. 2b,c). In many cases, the blebs detached from the membrane surface and resembled liposomes as previously observed with Xenopus oocytes^33^. Using a β-tubulin-GFP, we showed that β-tubulin is also absent (Supplementary Fig. 2)^36^.

### Blebs present a functionally different membrane environment

**Figure 3:**
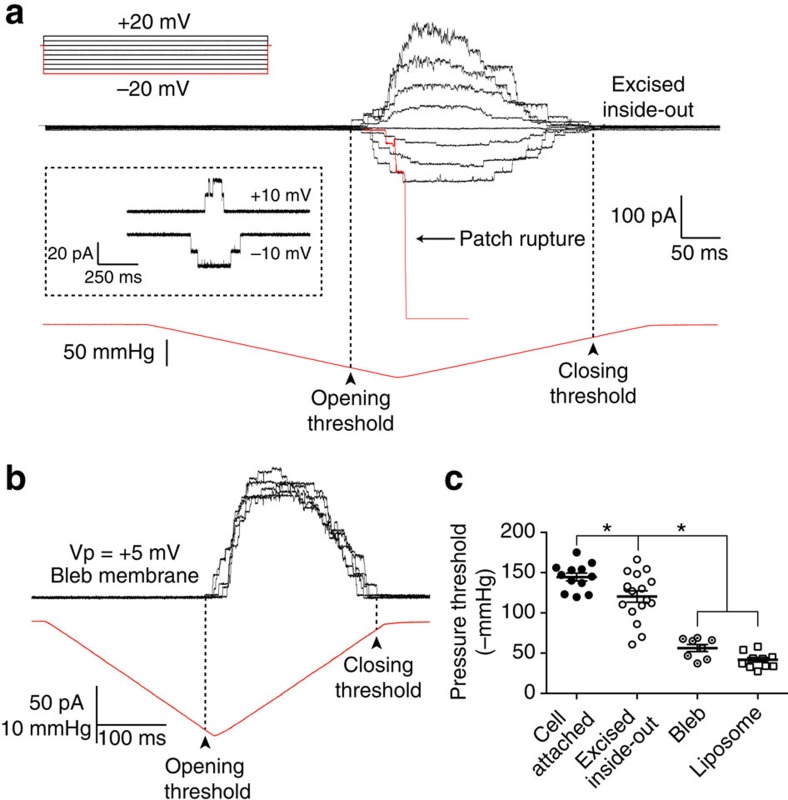
Activation of MscL–GFP in bleb-attached patches clearly illustrates the different mechanical environments. (**a**) A family of MscL current activity in an excised inside-out patch in response to pressure ramps (300 s to peak) at voltages ranging from +20 to −20 mV. (Inset shows single channel activity elicited by manual pressure application at two opposing voltages). In symmetrical NaCl, 145 mM MscL gives a conductance of 1.57 nS in excised patches. The conductance is somewhat lower than in previous reports owing to a reduction in the bulk conductivity of this recording solution in comparison with the widely used KCl 200 mM and MgCl_2_ 40 mM buffer. (**b**) Illustration of MscL activity in membrane blebs; traces show six sweeps of identical pressures. (**c**) Pressure thresholds of activation for WT MscL in four configurations; cell-attached, excised, bleb-attached and excised azolectin liposome (data points represent individual experiments with mean±s.e.m. shown for comparison). Pressure thresholds in cell-attached and excised were some three times higher than those seen in bleb-attached patches (*P value <0.01; one-way analysis of variance, Tukey's *post hoc* test).

To assess whether the blebs had a different mechanical environment for the channel than a cell-attached patch, we used the well-characterized prokaryotic mechanosensitive channel MscL. This channel has been extensively studied and is known to gate in response to bilayer tension^34^. We generated a carboxy (C)-terminal MscL fusion protein and characterized its electrophysiological properties and found that in cell-attached patches it activated with a pressure threshold of 144±5 mm Hg (*n*=12; ±s.e.m.; Fig. 3). The pressure threshold is defined as the pressure at which the first channel current appears. The threshold was higher in cell-attached than in inside-out patches where the cytoskeleton is more disrupted (120±8 mm Hg; *n*=17; Fig. 3a,c). The pressure threshold was also 30±6% (*n*=12) lower in excised patches where cell-attached activity as a reference was available before excision. The average number of channels per patch in excised patches was 13±3 (*n*=17). The pressure that gated the MscL–cGFP fusion protein was higher than reported for MscL in HEK cells (∼90 mm Hg)^37^. This is likely due to modulation by the fusion of GFP to the C-terminal since the same effect was observed in bacterial spheroplasts^38^. In blebbed membranes, MscL activated at 56±5 mm Hg (*n*=7), close to the values reported for gating in liposomes^39^ (Fig. 3b,c). Thus, the blebbed membrane is a different mechanical environment than the cell cortex and is more similar to liposomes rather than cell membranes. This emphasizes the mechanical influence of the cytoskeleton on plasma membrane stress and reaffirms the functional presence of the cytoskeleton in excised patches^13^,^23^,^40^.

**Figure 4:**
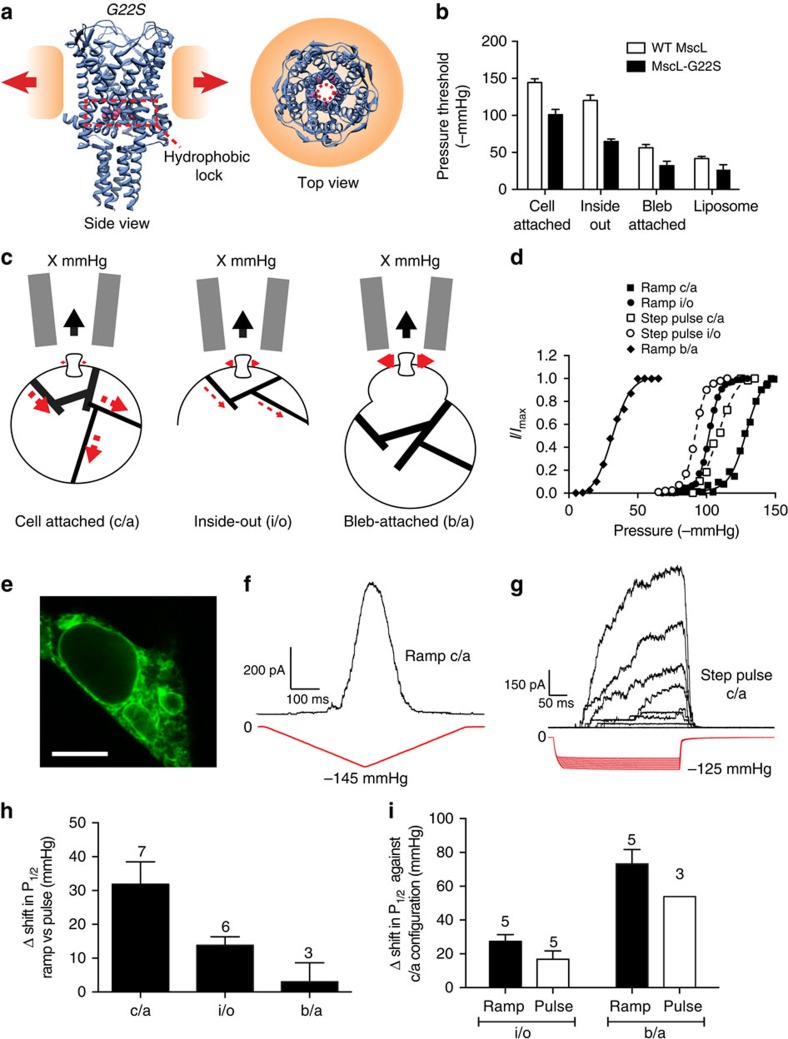
Activity of MscL–G22S–cGFP construct in cells provides further evidence for the lack of cortical cytoskeleton in bleb membranes. (**a**) Illustration of MscL structure with reference to the hydrophobic lock where the G22S mutation is housed. (**b**) Comparison of the activation pressures of the G22S mutation with the WT MscL channel in various configurations. (**c**) Graphic illustration indicating that there is a progressive loss of cortical cytoskeletal involvement from cell-attached to bleb-attached configuration with a corresponding reduction in the ability of the cytoskeleton to redistribute the applied force. (**d**) Activity of G22S–MscL–cGFP in the three configurations in response to either a ramp (350 ms to peak) or a square wave pressure pulse. A leftward shift in the Boltzmann distribution is obvious, not only between configurations but also between ramps and pulses, both applied via a high-speed pressure servo. (**e**) Confocal image of MscL–G22S–cGFP expressed in HEK293 cells. The channel is not confined to the plasma membrane and seems to be also incorporated in many organelle membranes (scale bar, 10 μm). (**f**) Representative trace showing G22S–MscL–cGFP activity in response to a pressure ramp up to a peak of −145 mm Hg (350 ms to peak). (**g**) Representative trace showing G22S–MscL–cGFP activity in response to a pressure pulse of 350 ms duration. (**h**) Quantification of leftward shift of *P*_1/2_ between ramp and pulse responses of G22S–MscL–cGFP. (**i**) Quantification of leftward shift of *P*_1/2_ between ramp and pulse responses of G22S–MscL–cGFP in comparison with the cell-attached patches (c/a, cell-attached; b/a, bleb-attached; i/o, inside-out; data points throughout represent mean±s.e.m.).

We further probed the environment using the more-sensitive MscL mutant, MscL–G22S–cGFP (Fig. 4) where a hydrophilic residue in the hydrophobic pore causes a reduction in the energy required for gating (Fig. 4a,b)^41^. This channel showed a similar reduction in the pressure threshold for activation in the different membrane environments (Fig. 4b). The G22S mutant enabled us to activate multichannel currents in the cell-attached configuration without rupturing the patch (Fig. 4b–i). Interestingly, the MscL–GFP fusion constructs were not only confined to the plasma membrane but were also present in the membranes of organelles (Fig. 4e).

We measured the gating curve in three patch configurations (Fig. 4c) and found a consistent leftward shift indicative of increased tension in the bilayer of blebbed membranes for a given amount of pipette suction. A similar shift was observed with ramp pressure protocols (Fig. 4d,f,g). Ramp protocols allow more time for a redistribution of force than a pulse and this produced a larger shift in the *P*_1/2_ for MscL–G22S–cGFP since stress in the cytoskeleton had more time to relax (Fig. 4h,i). This is depicted diagrammatically in Fig. 4c.

We further probed how mechanoprotection by the cytoskeleton affected the activity of MscL–G22S–cGFP by applying cytochalasin D (10 μM) and colchicine (10 μM) to HEK293 cells. In cells pre-treated with these cytoskeletal-disrupting agents, the pressure threshold was reduced in cell-attached and excised patches (Supplementary Fig. 3). As expected, the leftward shift (lower stress) in the gating curve elicited by excision was lost. Thus membranes treated with these compounds behave more like membrane blebs.

### PIEZO1–1591–GFP fusion protein behaves as WT

**Figure 5:**
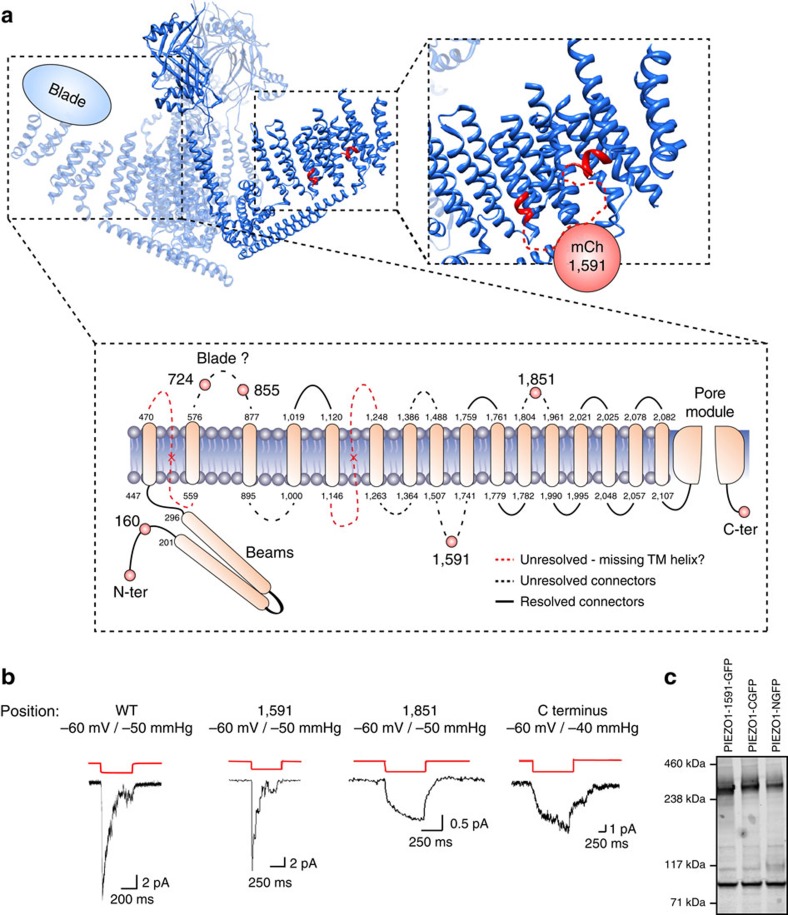
Activity of different PIEZO1 fluorescent constructs using GFP and mCherry. (**a**) Cryo-EM structure of mouse PIEZO1 with an inset showing the likely position of the 1591 insertion (mCherry or GFP)^47^. In addition, we show the putative topology of the transmembrane segments and the positioning of our fluorophore insertions using biochemical data^48^. (**b**) To create a fluorescent GFP–PIEZO1 protein, we first attached GFP to the N- and C-terminal end of the channel protein. The response of the channel was disrupted as it did not inactivate, and gave rise to small currents (trace labelled C terminus). We then introduced mCherry fluorescent protein into the predicted loop positions indicated in **a**. We were unable to observe any channel response from channels with mCherry insertions at positions 160, 724 and 855. (**b**) However, the addition of mCherry into position 1591 (called PIEZO1–1591–mCherry) was similar to WT with an inactivation time constant of 40 ms. The introduction of the fluorescent protein at position 1851 produced low currents and the channel did not inactivate. (**c**) A representative western blot showing three PIEZO1–GFP fusion constructs (N- and C-linked PIEZO–GFP and PIEZO1–1591–GFP) express full-length protein ∼300 kDa. Despite the lower number of channels encountered in the N- and C-terminal fusion proteins, total expression levels were similar. Lower band (∼100 kDa) shows α-actinin for comparison (*n*=3).

After characterizing MscL in blebbed membranes, we repeated the experiments using a fluorescently labelled PIEZO1 fusion construct. The necessity for a fluorescent construct in this situation stems from the fact that some proteins can be excluded from blebbing membranes^42^. With GFP at either the amino (N)- or C-terminal of PIEZO1, we found that in the cell-attached configuration, the channel kinetics showed delayed inactivation relative to controls. In addition, the channel number per patch for the N–GFP fusion was much lower than WT, despite the presence of comparable amounts of protein. This may be a result of abrogated trafficking (Fig. 5a–c).

**Figure 6:**
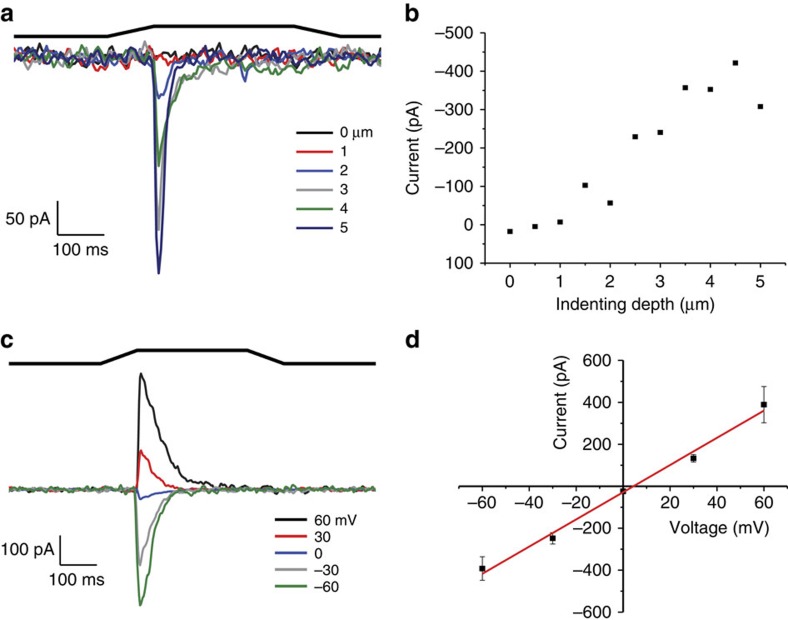
Whole-cell recording for PIEZO1–1591–mCherry on HEK293 cells produce robust currents. (**a**) Whole-cell currents elicited by pressing on the cell with a glass probe to the indicated depths. The membrane voltage was set to −60 mV. Above the current trace is the stimulus waveform. (**b**) Current plotted as a function of depth, which was incrementally increased at 0.5 micron intervals. Increased depth increases the current. (**c**) Currents recorded when the probe is set to single depth at varying membrane potentials. (**d**) Current as a function of voltage (from **c**) showing that the reversal potential is near 0 mV as in the wild-type PIEZO1 channel (data points represent mean±s.e.m.; *n*=5).

We then introduced mCherry or GFP into an internal site in an effort to maintain activity. We tested five internal sites that are putative loop structures (Fig. 5a,b). Surprisingly when we introduced mCherry to position 1591 (called PIEZO1–1591–mCherry), the kinetics were similar to the WT channel (Fig. 5b) with an inactivation time constant of ∼40 ms in cell-attached mode. The other mutants displayed abnormal activity, for example mCherry inserted at position 1851 provided a response similar to that observed for N- or C-terminal modification as it did not inactivate and had small currents (*n*=4, Fig. 5b). Three mutants (at positions 160, 724 and 855) produced no current (*n*=4). We confirmed that three of these constructs (N-terminal, C-terminal and 1591 insertion) expressed full-length protein by probing with a GFP antibody (Fig. 5c). The response of PIEZO1–1591–mCherry using whole-cell recording is shown in Fig. 6, and the response was similar to the WT channel with a reversal potential near 0 mV and robust mechanically induced currents.

**Figure 7:**
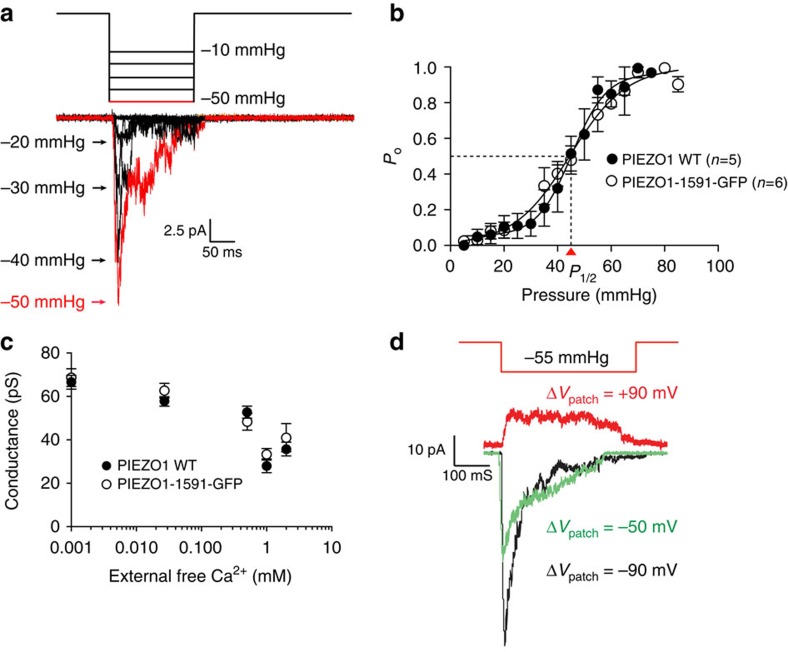
PIEZO1–1591–GFP fusion protein behaves similar to WT PIEZO1. (**a**) PIEZO1–1591–GFP currents elicited from a stepwise increase (−10 to −50 mm Hg) in pressure of 150 ms duration. The peak current generated from each pulse is labelled for clarity. (**b**) Open probability (*P*_o_) plotted against pressure pulse magnitude for PIEZO1–1591–GFP and WT PIEZO1. Here *P*_o_ is estimated as *I*/*I*_max_ from peak currents with increasing pressure pulses. (**c**) Effect of raising external Ca^2+^ concentration on single-channel conductance of PIEZO1–1591–GFP. Initial conductance in CsCl with ∼1 μM free Ca^2+^ is ∼62 pS (buffered using EGTA and calculated using Ca-EGTA Calculator v1.3, an online EGTA Ca^2+^ chelator calculator), and is reduced by almost half by 1 mM external Ca^2+^ (data points represent mean±s.e.m.; *n*=4). (**d**) Example of PIEZO1–1591–GFP voltage-dependent inactivation at three voltages. Inactivation markedly slows with depolarization as seen in WT PIEZO1 (for comparison with previous results: Δ*V*_patch_=−55 mV; *τ*=55±11 ms, *n*=6).

The PIEZO1–1591–GFP construct also behaved similarly to wild-type channels including a reduction in unitary conductance with increasing external Ca^2+^ and voltage-dependent inactivation (Fig. 7a–d)^5^,^43^,^44^. The kinetics of inactivation gave time constants similar to those previously reported for whole-cell mode (Δ*V*_patch_=−55 mV: *τ*=55±11 ms; *n*=6). The pressure sensitivity in cell-attached patches gave a *P*_1/2_ of 44±2 mm Hg (*n*=6), almost identical to the WT PIEZO1 channel *P*_1/2_ of 45±3 mm Hg (*n*=5). The slope sensitivity (the slope of the *P*_open_ curve versus pressure at the midpoint, 1/α) was also similar to the WT (0.10±0.02 mm Hg^−1^ and PIEZO1–1591–GFP fusion; 0.15±0.04 mm Hg^−1^). The channel could also be gated by positive pressure in the cell-attached configuration as seen for other mechanosensitive channels gated by membrane tension^17^,^45^,^46^ (Supplementary Fig. 4). In the following experiments, we used channels with the non-perturbing insertions of mCherry or GFP.

Recently the cryo-EM structure of murine PIEZO1 was solved and shown to be a trimer and along with biochemical data it provides structural clues to the functional effects that we saw upon fluorophore insertion (Fig. 5)^47^,^48^. For example, positions 724 and 855 where insertions give rise to non-functional channels appear to be in an extracellular region termed the ‘blades', which have been suggested to be important in the sensing of mechanical stimuli. Both biochemical data and cryo-EM data suggest that a fluorophore inserted at position 1591, is likely to be intracellular and arranged close to the periphery of each ‘wing'. This gives a plausible reason as to why such large insertions can result in channel activity almost indistinguishable from WT.

### Visualizing the fluorescent PIEZO1 constructs in HEK293 cells

With PIEZO1–1591–mCherry (250 ng) and TREK-1–GFP (50 ng) co-transfected into HEK-293 cells, images made with Structured Illumination Microscopy (SIM-API) (Supplementary Fig. 5) show that the two mechanical channels are in separate domains of differing sizes. In some regions of the cell, TREK-1 appears in bead-like chains suggesting that they are organized along cytoskeletal fibres. The association of TREK-1 to the underlying actin cytoskeleton has been noted previously^46^.

### PIEZO1 in bleb membranes

**Figure 8:**
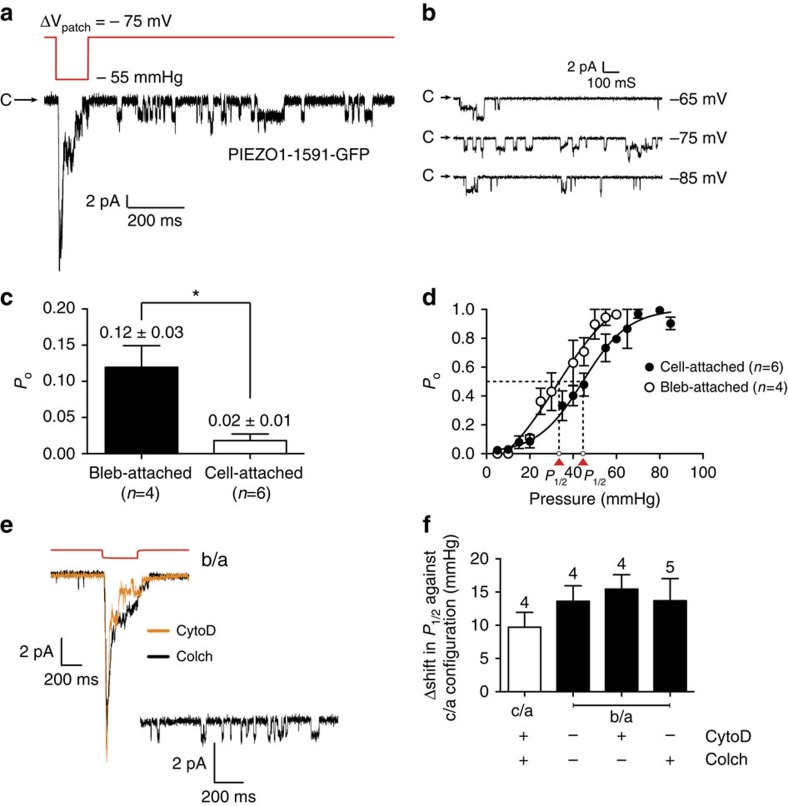
Activation of the PIEZO1–1591–GFP fusion protein in bleb-attached patches in comparison with cell-attached. (**a**) Mechanosensitivity of PIEZO1–1591–GFP in bleb-attached patches in response to a 150 ms square wave pressure pulse of −55 mm Hg (pipette: CsCl). (**b**) Single-channel activity at rest as seen post pressure at three different voltages. (**c**) The increase in *P*_o_ at zero pressure is calculated using 5 s records. There is at least a fivefold increase in basal activity (*P<0.01; Student's *t*-test). (**d**) Leftward shift in Boltzmann distribution function in bleb-attached patches (cell-attached *P*_1/2_=44±2 mm Hg; *n*=6: bleb-attached *P*_1/2_=33±3 mm Hg; *n*=4). (**e**) Example traces of PIEZO1–1591–GFP activated in blebbed membranes pre-treated with Cytochalasin D (CytoD) 10 μM and colchicine (Colch) 10 μM. Cells were treated for 1 h pre-blebbing, then blebbed in the presence of these agents for 2 h using a hypo-osmotic solution. Final patch-clamp electrophysiology was carried out immediately with the bath solution again containing the same pharmacological agents. (**f**) Quantification of leftward shift of *P*_1/2_ between cell-attached patches and bleb-attached patches with the identified cytoskeletal interfering agents (c/a, cell-attached; b/a, bleb-attached; data represents mean±s.e.m.; *n*=4/5).

We activated PIEZO1–1591–GFP in bleb-attached patches (Fig. 8a) and quantitative analysis showed that the basal activity was higher than that in cell-attached patches indicative of pretension resulting from gigaseal formation^15^. While ‘gating at rest' is often encountered in cell-attached patches, it is not as pronounced as in bleb-attached patches (Fig. 8a–c). The adhesion energy of membranes to the glass pipette creates baseline tension and this tension background is common to all patches^13^. As seen with MscL, channel activation in blebs showed a leftward shift in the gating curve (*P*_1/2_=33±3 mm Hg, *n*=4) relative to cell-attached patches. The slope sensitivity (0.15±0.04 mm Hg^−1^) was the same as the WT suggesting the closed–open conformational change was the same (0.18±0.05 mm Hg^1^; Fig. 8d).

To further compound the lack of cortical cytoskeleton in the blebbed membranes, we pre-treated cells with cytochalasin D (10 μM, *n*=4) and colchicine (10 μM, *n*=5) to see whether the activity of PIEZO1–1591–GFP was modified (Fig. 8e). Treatment with neither agent modified the gating curve implying that bleb formation disrupts most of the structural cytoskeleton (Fig. 8f). Consistent again with MscL, and as further evidence of mechanoprotection in the cell-attached patches co-treatment of cell-attached patches with both cytochalasin D (10 μM) and colchicine (10 μM) reduced the activation pressures for PIEZO1–1591–GFP (Fig. 8f).

### Estimating the tension sensitivity of gating PIEZO1

**Figure 9:**
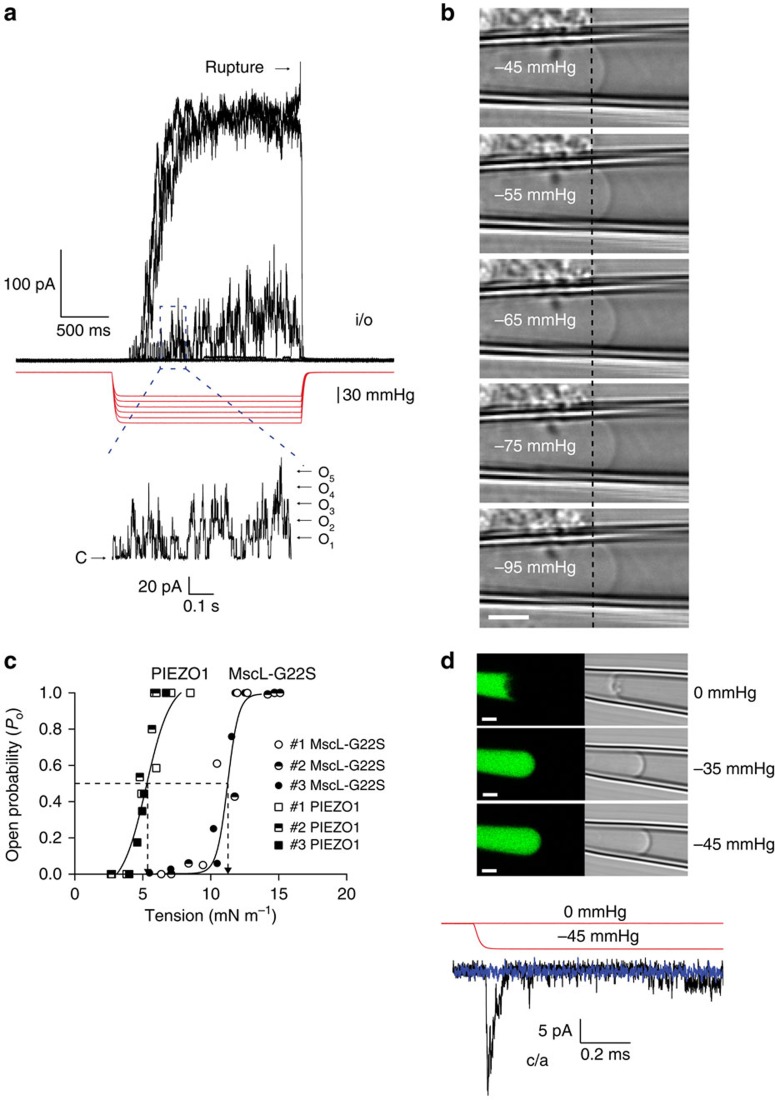
Estimating the tension required to gate PIEZO1 channels. (**a**) Family of MscL–G22S–cGFP channel currents elicited in an excised inside-out patch at +10 mV pipette potential. Inset shows enlargement of a segment of one sweep clearly documenting single MscL channel transitions. (**b**) Confocal images of the corresponding patch membrane and its deformation over time under the negative pressures. The images corresponding to the pressure steps seen in **a** allow calculation of membrane tension using Laplace's law (*T*=*Pr*/2; where *T* is tension, *P* is the applied pressure and *r* is the radius of patch curvature). Laplace's law provides an upper limit for the tension sensitivity. (**c**) A Boltzmann distribution is shown for three independent experiments illustrating how membrane tension is linked to channel open probability. The same analysis is shown for PIEZO1 using data accrued from the cell-attached patches as shown in **d**. The fluorescence is that of GFP being expressed on the same plasmid as used to deliver the WT PIEZO1 channels. The patch-clamp recording below the visualized patch membranes shows the corresponding currents elicited by membrane deformation (blue, 0 mm Hg; black, −45 mm Hg; Δ*V*_patch_=−55 mV; scale bar, 2 μm in all the images).

Thus far, we have shown that PIEZO1 can be gated in bleb-attached patches in the absence of the cytoskeleton from which we can strongly suggest that the channel is gated by bilayer tension. The next question is what is the absolute tension sensitivity of PIEZO1? We co-transfected MscL–G22S–cGFP and PIEZO1 and measured the *P*_1/2_ for gating (*P*_1/2_ is the pressure at which *P*_open_=1/2 of saturation). The ratio of *P*_1/2_ was ∼0.4, that is, the *P*_1/2_ of WT PIEZO1 was 40% of the *P*_1/2_ for MscL–G22S–cGFP in cell-attached patches (Supplementary Fig. 6). We examined the tension required to gate MscL–G22S–cGFP in excised inside-out patches that are easily imaged using confocal microscopy. We activated MscL–G22S–cGFP with 2 s pulses of negative pressure of increasing magnitude and monitored the corresponding patch deformation (Fig. 9a,b) allowing us to use Laplace's law to determine mean tension in the patch dome^13^,^23^. Under these conditions, the channels began to gate at ∼8.5–10 mN m^−1^ with a *T*_1/2_ (tension where *P*_o_=0.5) of 11.3±0.6 mN m^−1^ (*n*=3, Fig. 9c). This corresponds to a free energy of gating, Δ*E*=40±4 kT (*n*=3, the energy required to open the channels with no applied tension) which corresponds to an in-plane area change (Δ*A*) of 15±2 nm^2^. This large expansion is characteristic of mechanosensitive channels. From the co-expression data, we estimate that the tension at *P*_1/2_ is ∼4.5 mN m^−1^ for PIEZO1.

To validate this estimate, we expressed PIEZO1 channels in HEK293 cells with a bicistronic plasmid also expressing GFP to sharpen the cell boundaries (Fig. 9c–e). In the cell-attached configuration, *T*_1/2_ of PIEZO1 was 5.1±0.2 mN m^−1^ (*n*=3, Fig. 9c–e), and Δ*E*=9.7±1.5 kT (*n*=3) with Δ*A*=8±1 nm^2^.

## Discussion

Our experimental strategy of generating membrane blebs has followed the study of mechanosensitive channels by Zhang *et al.*^33^, using Xenopus oocytes^30^,^31^. The blebs are clearly deficient in cytoskeletal components including f-actin and β-tubulin^25^,^26^. As an internal calibrator of tension, we used MscL that has been calibrated in liposomes. The pressure sensitivity in bleb membranes was threefold less than cell-attached patches and is close to that seen in liposomes^39^. This demonstrates the protective effect of the cytoskeleton on membrane-embedded proteins, that is, ‘mechanoprotection' and emphasizes that any intervention that alters cytoskeletal structure is likely to affect channel gating^12^,^13^,^49^,^50^. MscL channels in excised inside-out patches from cells required more pressure (2.5 times the pressure) to gate than in blebs. Excision interferes with, but does not completely disrupt tension sharing by the cytoskeleton^51^,^52^,^53^. This is supported by pretreatment with colchicine and cytochalasin D, which also reduced the force required to gate MscL in both cell-attached and excised configurations. The imaging of fluorescent PIEZO1 and TREK-1 showed that the two channel types are segregated to separate domains. A critical consequence is that the tension within each domain may be different since the line tension of the domains requires that the internal tension is different from external tension^54^. An interesting feature of the TREK-1 distribution was an apparent alignment of some channels to underlying linear cytoskeletal elements. This interaction is supported by the observation that a non-conducting point mutation in TREK-1 has a dramatic effect on the cytoskeletal reorganization^46^. Although these data do not unequivocally support the idea that PIEZO1 resides in a ‘cytoskeleton free' domain, they do illustrate that PIEZO1 resides in a different cellular domain to TREK-1. This is interesting and fits with the proposed functional crosstalk between these two types of channels^17^. However, ultimately TREK-1 as well as the related protein TRAAK, sense force transmitted directly through the lipid bilayer despite their previously affirmed association with the cytoskeleton^16^,^17^. If PIEZO1 channels were gated by bilayer tension, like TREK-1, TRAAK^16^,^17^, MscL and MscS-like channels^55^, we would expect to see a similar trend in its activity when compared with MscL in bleb-attached patches, and this is what is found. PIEZO1 can be activated in bleb membranes with less applied pressure than in cells. Furthermore, this is unaffected by interfering with either actin polymerization (CytoD) or microtubule polymerization (colchicine) providing further support for an absence of cytoskeletal linkages in blebbed membranes. However, if PIEZO1 is gated by bilayer tension, an obvious question that arises is: ‘does purified PIEZO1 protein exhibit mechanosensitive gating when reconstituted?'^2^. The answer thus far seems to be no and there are a number of possible explanations for this. The data from planar bilayers do not exhibit inactivation kinetics^2^. This could arise because the data actually came from occasional channels returning from the inactivated (tension insensitive) state since the resting tension in bilayers is on the order of 4–6 mN m^−1^. Alternatively, as others have reported, we saw rapid rundown of PIEZO1 channel currents in the excised inside-out configuration, and recent work has shown that this rundown is correlated with a loss of phosphoinositides, in particular PIP2 (ref. 56). The lack of PIP_2_ in reconstituted bilayers may explain the lack of response to applied membrane tension documented in this environment^2^. The fact that PIEZO1 channels can be activated in blebs virtually eliminates the possibility that activation requires binding to the cytoskeleton and biochemical data have shown no specific association of PIEZO1 with other proteins^2^. Future work should aim to address the question as to why does purified PIEZO protein not exhibit mechanosensitive gating when reconstituted; answering such a central question will no doubt uncover important functional information.

It is important to note that our work does not preclude modulation by structural proteins of the cytoskeleton or extracellular matrix. For example, PIEZO1 is sensitized by the expression of STOML3, a cytoskeleton-associated protein^57^, and this may well alter the force distribution to the bilayer^49^. In fact we have already shown that PIEZO1 currents elicited by cell indentation are markedly reduced by pre-treatment with cytochalasin D. This, however, is a result of force transmission in this configuration and not a direct link to the channel as when we pre-stressed the bilayer with hypo-osmotic shock in the presence of cytochalasin D, the currents were boosted^43^.

Here we have provided the first direct assessment of the tension sensitivity of PIEZO1. The first method utilized the gating of MscL–G22S–cGFP channels in inside-out patches (*T*_1/2_=11.3±0.6 mN m^−1^; *n*=3). We then used this value to estimate the tension required to gate PIEZO1 channels from the pressures required to gate PIEZO1 when co-expressed with MscL–G22S–cGFP. This gives a *T*_1/2_ of ∼4.5 mN m^−1^. We confirmed this by imaging the patch geometry and estimated *T*_1/2_=5.1±0.2 mN m^−1^ (*n*=3). Applying Laplace's law to heterogeneous three-dimensional structures is a simplification of the complex material properties of a cell^13^,^58^, but it does provide a useful upper limit of the magnitude of the forces required to gate the channel. Furthermore, an inherent characteristic of mechanosensitive channels is that they undergo large in-plane area expansion (Δ*A*) during gating allowing the tension field to do work on the channel. From the tension and the free energy difference between the closed and open states, we estimated Δ*A*∼15 nm^2^ for MscL–G22S–cGFP fitting well with previous estimates^59^,^60^. PIEZO1 also appears to undergo a large in-plane expansion of ∼8 nm^2^ (ref. 5). These calculations assume that the free energy of gating is driven purely by a change of in-plane area and that may not be completely accurate^61^.

The quantification of PIEZO1 gating may be used as an internal calibrator of how efficiently a stimulus can reach the channel. Our data also emphasize that drug screens of PIEZO channels are predicted to be sensitive to cytoskeletal modulation and lipid modulation that can change the distribution of stresses between the bilayer, the cytoskeleton and the extracellular matrix. Collectively, these results illustrate the utility of prokaryotic mechanosensitive channels as models and tools for studying mechanosensory transduction, and the wide applicability of basic biophysical principles in the gating of mechanosensitive channels irrespective of their evolutionary provenance.

## Methods

### PIEZO1 and MscL GFP fusion constructs

*Construction of C- and N-terminally labelled PIEZO1*. Two vectors, pNGFP-EU and pCGFP-EU (gift of Eric Gouaux), were used to introduce an EGFP-fusion label at either the N- or C-terminal of *PIEZO1*, respectively. For pNGFP-EU, *PIEZO1* was inserted between the HindIII and BamHI sites. To achieve this, we amplified the gene with a forward primer having a HindIII restriction site and a reverse primer having a BamHI site with Prime star GXL DNA polymerase (Clontech/Takara). The DNA was purified by ZymoResearch Clean Kit according to manufacturer's specifications. The vector pNGFP-EU was treated with BamHI and HindIII restriction endonuclease and purified by ZymoResearch Clean Kit. *PIEZO1* DNA (100 ng) and treated vector (30 ng) were assembled by InFusion (Clontech/Takara). Stellar cells were transformed after 15 min. Colonies were analysed by restriction analysis and then by sequencing.

A similar protocol was used to covalently attach EGFP to the C-terminal of PIEZO1. We amplified *PIEZO1* with primers carrying HindIII and EcoRI restriction sites as described above. pCGFP-EU was prepared by treatment with HindII and EcoRI restriction endonucleases. The vector and *PIEZO1* was assembled by InFusion as described above.

*Construction of internally labelled PIEZO1*. We inserted the fluorescent protein, mCherry, into five internal sites at amino acid positions 160, 724, 855, 1591 and 1851. This was achieved by first introducing Age1 and Spe1 sites into DNA encoding PIEZO1 at the indicated positions using QuikChange II XL Mutagenesis kit (Agilent).

All clones were analysed by first isolating plasmid DNA using the ZymoResearch mini prep kit according to the manufacturer's specification and restriction endonuclease digestion. To insert DNA coding for mCherry fluorescent protein into each Age1/Spe1 site, we amplified the DNA for the mCherry protein with primers that added Age1 (forward) and Spe1 (Reverse) restriction sites at the ends.

Plasmid and mCherry PCR products were cut with Age1 and Spe1 and then purified. Ligation of the mCherry DNA and each plasmid (3:1 or 10:1 concentration) was achieved using Quick Ligase, (New England Biolabs). Each reaction was transformed into Stellar cells with indicated antibiotic for selection. The plasmid DNA was purified and sequenced.

The expression of EGFP from the pIRES vector was silenced by a frameshift mutation using Agilent's QuikChange II XL kit with 100 ng of template DNA and with an extension time of 21 min.

To replace the mCherry protein with the GFP fluorescent protein at position 1591, we used the above primers and amplified the DNA from the pIRES-EGFP vectors. The Plasmid PIEZO1–1591–mCherry was cut with Spe1 and Age1 to remove the DNA that expresses the mCherry protein and purified by gel electrophoresis. The amplified DNA for GFP was treated with Spe1 and Age1 and purified. The final products were ligated and transformed (3–4 μl) into Stellar cells and the plasmid DNA isolated and sequenced.

MscL with C-terminal-fused EGFP constructs were cloned by PCR, using PfuUltra (Agilent), for insertion into pTRE-Tight (Clontech) EcoRI and HindIII restriction sites. The MscL forward primer contained the following Kozak sequence 5′-gccAccATGGcg-3′ after the EcoRI site, which consequently inserted an alanine following the MscL methionine 1 ATG start site. An EGFP with an N-terminal thrombin cleavage site construct contained within the laboratory was used as the PCR template for EGFP. The reverse MscL primer, and the forward thrombin-site EGFP primer both contained an in-frame NheI restriction site that was utilized to ligate MscL to EGFP while both inserts were ligated together into pTRE-Tight. MscL mutants were cloned by the same method using a previously made mutant (MscL-G22S) contained within the laboratory as MscL PCR template. The final pTRE-Tight MscL–T–eGFP construct insertions were sequence verified. Plasmid maxipreps were prepared using a PureLink kit (Life Technologies), with a NucleoBond Finalizer (Macherey–Nagel) using 1 ml TE for elution.

### Transient transfection

HEK293 cells were transiently transfected with 250–650 ng of cDNA using Lipofectamine 3000 in OptiMEM. Electrophysiological analysis was undertaken 24–72 h post transfection.

### Bleb formation

Blebs of HEK293 cells were formed using a modified Ca^2+^ free KCl solution (in mM: 140 KCl, 10 EGTA, 1 MgCl_2_, 15 HEPES pH 7.2 adjusted with KOH)^35^, hyperosmotic sodium gluconate solution (∼440 mOsm) or a hypo-osmotic sodium gluconate solution (∼140 mOsm). Sodium gluconate solutions were diluted with ddH_2_O from stock containing in mM: 350 sodium gluconate, 2 CaCl_2_, 3 KCl, 15 glucose and 20 HEPES pH 7.2, adjusted with NaOH. Blebs took between 45 min and 2 h to appear depending on the solution used. For electrophysiological recordings, cells were plated out onto 35 mm circular dishes (FluoroDish, World Precision Instruments, Inc) coated with poly-L-lysine and were treated for 3 h using a hypo-osmotic solution to induce blebbing. After this, blebs were directly patched using the recording solutions mentioned for up to 45 min. No bleb retraction was seen during this period of time.

### Liposome reconstitution

MscL proteoliposomes were prepared using the dehydration/rehydration method. This methodology is a modified procedure from^39^,^45^,^62^. WT MscL for reconstitution was purified using immobilized metal affinity chromatography^63^. MscL-G22S protein for reconstitution was expressed by cell-free expression using an automated system (Exiprogen, Bioneer, Daejeon, Korea) that also performed the protein purification steps after expression. Ten micrograms of expression plasmid was added to the kit used (EC-1, Bioneer), with a previously used detergent combination of 6 mM Brij-58 detergent (Sigma) added to the expression well, and 1% n-Octyl-β-D-glucopyranoside (OG) (Anatrace) added to the binding/wash buffer wells, as well as the elution buffer well^64^. Protein concentration was estimated by SDS–polyacrylamide gel electrophoresis (SDS–PAGE) with staining using SimplyBlue SafeStain (Life Technologies).

### Patch-clamp recording

Transiently transfected HEK293 cells were plated on coverslips for patch-clamp analysis at a density of ∼3,000 cells per coverslip. Coverslips were placed in a recording chamber containing 145 mM NaCl, 3 mM KCl, 1 mM MgCl_2_ and 10 mM HEPES (pH 7.2) adjusted using NaOH. In cell-attached and bleb-attached recordings, the pipette solution contained either 145 mM CsCl or 145 mM NaCl with 10 mM HEPES (pH 7.2) adjusted using the respective hydroxide. EGTA was added to control levels of free pipette (extracellular) Ca^2+^ using an available online EGTA calculator—Ca-EGTA Calculator TS v1.3—Maxchelator. Negative pressure was applied to patch pipettes using a High Speed Pressure Clamp-1 (ALA Scientific Instruments) or recorded in millimeters of mercury (mmHg) using a piezoelectric pressure transducer (WPI, Sarasota, FL, USA). Borosilicate glass pipettes (Sigma, St Louis, MO, USA) were pulled using a vertical pipette puller (PP-83, Narashige, Japan) to produce electrodes with a resistance of 3.5–5.0 MΩ and coated with Sylgard to within ∼100 μm of the tip. Single-channel PIEZO1 currents were amplified using an AxoPatch 200B amplifier (Axon Instruments), and data were acquired at a sampling rate of 10 kHz with 1–2 kHz filtration and analysed using pCLAMP10 software (Axon Instruments). Boltzmann distribution functions shown in Figs 4, 6 and 9 describe dependence of single MscL and PIEZO1 channel currents and open probability, respectively, on the negative pressure applied to patch pipettes. The Boltzmann plots were obtained by fitting open probability *P*_o_∼*I*/*I*_max_ versus negative pressure using the expression *P*_o_/(1–*P*_o_)=exp [*α*(*P*–*P*_1/2_)], where *P* is the negative pressure (suction) [mmHg], *P*_1/2_ is the suction at which *P*_o_=0.5, and *α* [mmHg^−1^] is the slope of the plot ln [*P*_o_/(1–*P*_o_)=[*α*(*P*–*P*_1/2_)] reflecting the channel mechanosensitivity^65^.

The membrane tension *T* was estimated using Laplace's law (*T*=*Pr*/2) by measuring the radius of membrane patches *r* at applied pressures *P* (For a more detailed explanation, see supplement of ref. 66).

### Whole-cell recording

In whole-cell recording mode, cells were mechanically stimulated by pressing on the cell with a fire-polished glass pipette (diameter of 2–4 μm) positioned at an angle of 30° with respect to the cover glass^5^. The probe was coarsely positioned ∼20 μm above the cell with a MP-285 manipulator (Sutter Instruments Co.), and from that position, the probe was moved up and down with a trapezoidal waveform by a piezoelectric stage (P-280.20 XYZ NanoPositioner, Physik Instrumente). Control of the depth was controlled by LabVIEW software with 40 nm resolution. The probe velocity was constant at 0.15 μm ms^−1^ during transitions, and the stimulus was held constant for 300 ms. All currents were recorded at room temperature. The experiments were performed using an Axopatch 200B amplifier (Axon Instruments) sampled at 10 kHz and filtered at 1 kHz. Data acquisition and stimulation were all controlled by QUBIO software.

### Confocal microscopy

Cells were grown on 35-mm-diameter cell culture dishes (FluoroDish, World Precision Instruments, Inc) coated with poly-L-lysine (Sigma, 5 μg cm^−2^) and subjected to blebbing solutions identical to those used for electrophysiological study. Cells were then fixed for 15 min using 4% paraformaldehyde at room temperature. Subsequently, control cells or those expressing PIEZO1 or MscL GFP fusion proteins, were permeabilized using 0.5% Triton X-100 (Sigma) for 2 min and stained for 20 min at room temperature with Alexa Fluor 568 conjugated phalloidin (Sigma). Confocal images were made using an inverted confocal microscope (LSM 700; Carl Zeiss) equipped with a water immersion long working distance objective (× 63; NA 1.15; Carl Zeiss). Both 488 and 555 nm lasers were used to excite the respective fluorophores (GFP, Alexa Fluor 568).

### SIM microscopy

Plasmids for PIEZO1–1591–mCherry and TREK-1–EGFP were transfected into HEK293 cells, and allowed to incubate for 24 h. Cells were then washed twice with phosphate-buffered saline (PBS) and fixed with a 4% solution of paraformaldehyde in PBS for 30 min. After removal of the fixing solution, cells were washed and mounting media, Prolong Gold (Life Technologies), was applied to the cells. These were subsequently inverted and sealed on a glass coverslip. Data were collected by SIM (API, Seattle) with a × 60 objective NA 1.4 oil. Data were collected (24 z-stack) and reconstructed into a three-dimensional model using Vaad3D software^67^.

### Western blotting

HEK293 cells were transiently transfected in 24-well plates using Liposome-based transfection reagent Lipofectamine 3000 (Life Technologies, Carlsbad, CA, USA) to deliver PIEZO1 cDNA into the cells. Cells were lysed in a lysis buffer containing 1% NP-40 (Sigma-Aldrich, Castle Hill, NSW, Australia) and protease inhibitors (Roche, Cromer, NSW, Australia) after 48 h and equal volume of lysate loaded for SDS–PAGE and western blot analysis. The nitrocellulose membranes were probed simultaneously with a rabbit polyclonal anti-GFP antibody at a 1:5,000 dilution (Abcam, Cambridge, UK; Cat no—ab290) and a mouse monoclonal anti α-actinin antibody at 1:1,000 dilution (Santa Cruz Biotechnology, Dallas, TX, USA Cat no—sc17829) overnight. Both anti-mouse IRDye800 and anti-rabbit IRDye680 (Li-Cor Biotechnology, Lincoln, NE, USA) at a 1:20,000 dilution were incubated with the membrane and the PIEZO1 proteins were detected using the Li-Cor Odyssey system (Li-Cor Biotechnology). Western blot images were produced using ImageStudioLite (Li-Cor Biotechnology).

## Additional information

**How to cite this article:** Cox, C. D. *et al.* Removal of the mechanoprotective influence of the cytoskeleton reveals PIEZO1 is gated by bilayer tension. *Nat. Commun.* 7:10366 doi: 10.1038/ncomms10366 (2016).

## Supplementary Material

Supplementary Information Supplementary Figures 1-6
