# Supplementary material for: Removal of the mechanoprotective influence of the cytoskeleton reveals PIEZO1 is gated by bilayer tension
Source: Nat Commun. 2016 Jan 20;7:10366. doi: 10.1038/ncomms10366 (PMC4735864; doi:10.1038/ncomms10366)
Supplement: Supplementary Information — Supplementary Figures 1-6 [file ncomms10366-s1.pdf]

Supplementary Figures.

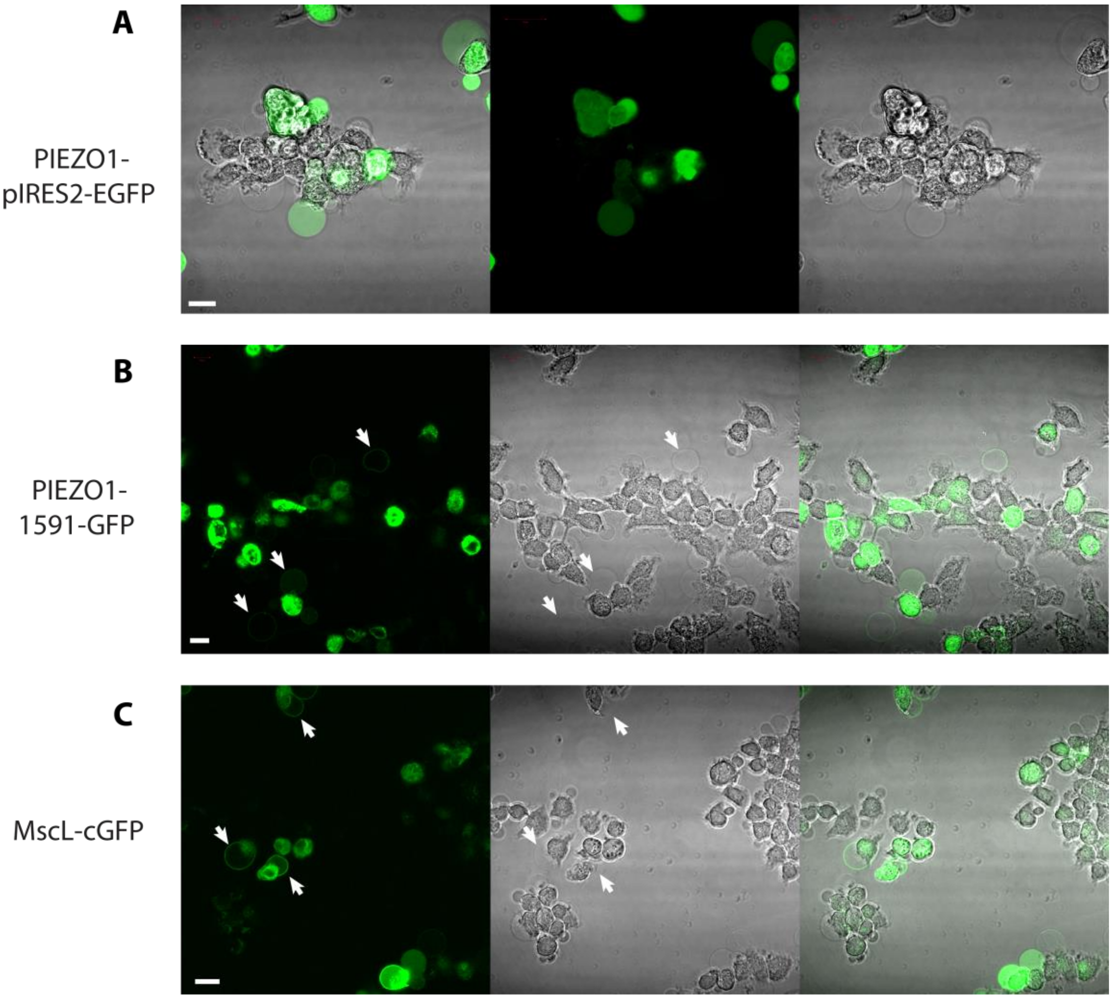

Supplementary Figure 1. Images showing a large number of transfected HEK293 cells with numerous blebs. (A) PIEZO1-pIRES2-EGFP **(B)** PIEZO1-1591-GFP (wider field from that shown in Fig. 2 main text) **(C)** MscL-cGFP (wider field from that shown in Fig. 2 main text) expressing HEK293 cells induced by hypo-osmotic NaGluconate solution. GFP fluorescence is clearly present in the bleb membranes indicated by the white arrows (bar represents 10 μm).

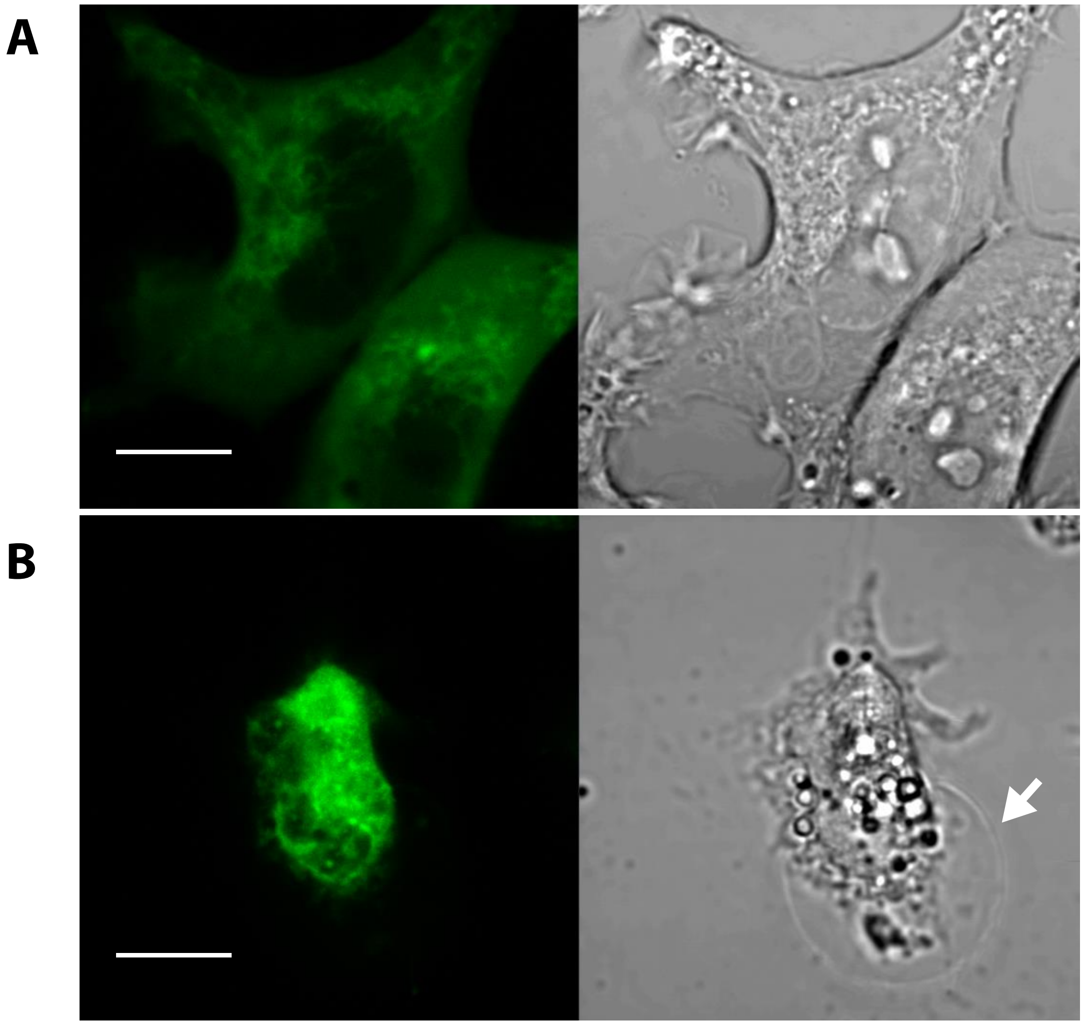

Supplementary Figure 2. β-tubulin is absent from bleb membranes in HEK293 cells. (A) Expression of β-tubulin-GFP construct (pd2EGFP-N1 HM40, Prof Maria Kavallaris, University of Sydney, Australia) in HEK293 cells. **(B)** GFP fluorescence is clearly absent from blebs indicated by the white arrow.

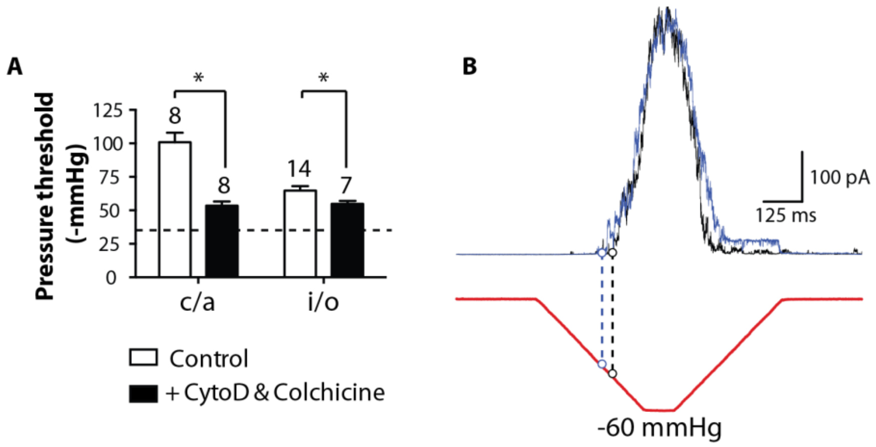

Supplementary Figure 3. The effect of CytoD and colchicine on the pressure threshold of MscL-G22S-cGFP. (A) Pressure thresholds of MscL-G22S-cGFP in cell-attached (c/a) and excised inside-out configuration (i/o) with and without pre-treatment with Cytochalasin D (CytoD) 10 µM and colchicine 10 µM. Pre-treatment was carried out for one hour before patching and the bathing solution contained both compounds to prevent any diminishing of the effect. Data represents mean ± SEM. Number above represents n of each group (*represents statistically significant difference, Student's T-test; $p<0.05$). **(B)** Example traces of MscL-G22S-cGFP activity in a cell-attached patch (two identical pressure ramps) after treatment with Cytochalasin D (CytoD) 10 µM and colchicine 10 µM for one hour showing the low pressure threshold of activation compared to control.

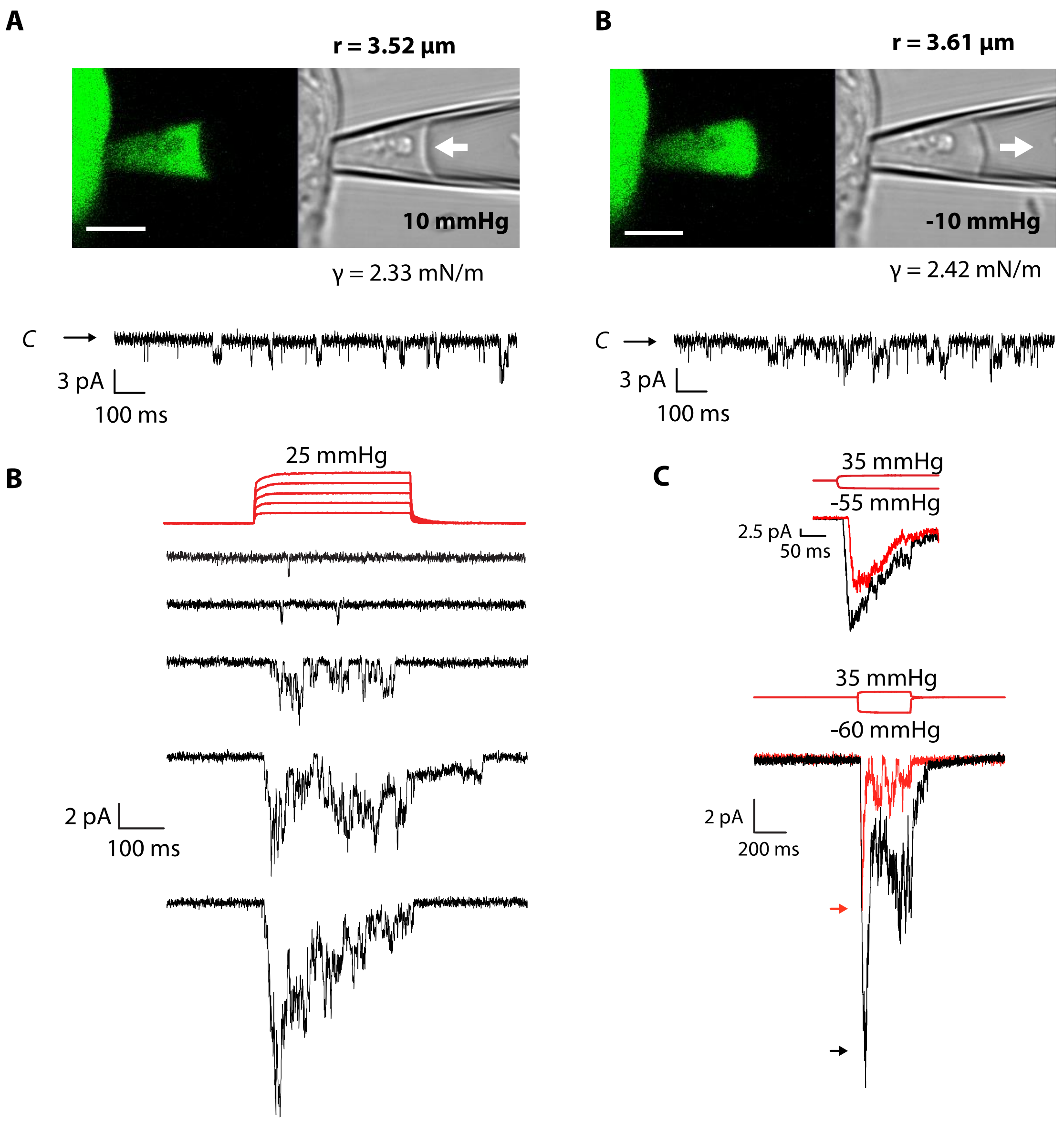

Supplementary Figure 4. Gating PIEZO1 using positive pressure. (A) Confocal image of a cell-attached patch of a HEK293 cell expressing WT PIEZO1 with 10 mmHg positive pressure (left panel) or -10 mmHg pressure (right panel) applied for 350 ms. The tension estimated using Laplace's law is shown along with the PIEZO1 activity concurrently recorded (below) at ΔV_{patch} = +65 mV. This tension fits well with the Boltzmann distribution shown in the Figure 9 (*represents statistically significant difference, Student's T-test; $p<0.05$). **(B)** Activity of PIEZO1 in a cell-attached patch in response to escalating positive pressure (5 mmHg to 25 mmHg) at ΔV_{patch} = +65 mV. **(C)** PIEZO1 activity in two cell-attached patches in response to positive and negative pressure pulses. In cell-attached configuration it was difficult to apply more than +35 mmHg. The records show the discrepancy between the current seen at positive vs negative pressure pulses (ΔV_{patch} = +95 mV).

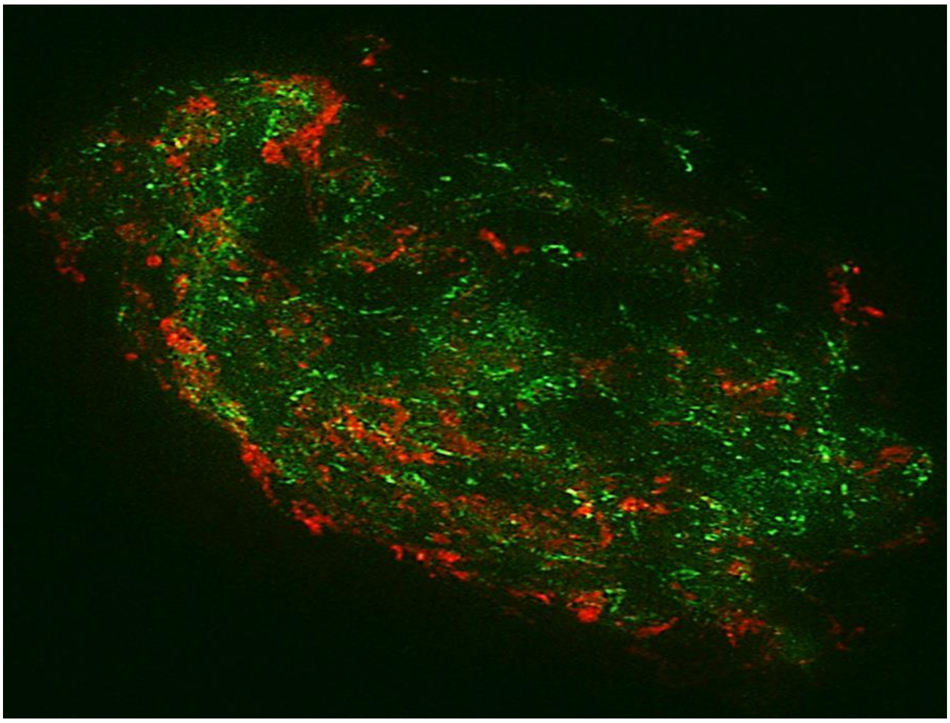

Supplementary Figure 5. 5PIEZO1 channels form clusters. Image of fluorescent proteins PIEZO1 (red) and TREK-1 (green) on the plasma membrane of HEK293 cells. PIEZO1 is tagged with internal mCherry and TREK-1 is linked to GFP through its C-terminus. TREK-1 forms bead like structures that appear to track an underlying cytoskeletal structure. In comparison, PIEZO1 forms larger cluster and there is no significant overlap of the two channels.

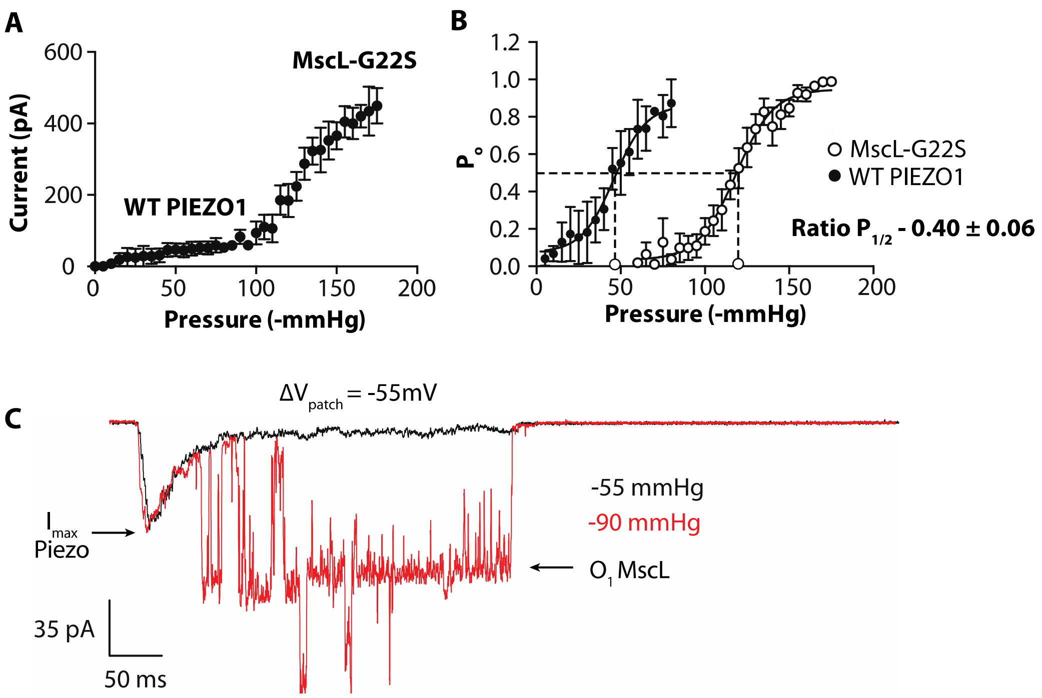

Supplementary Figure 6. Co-expression of PIEZO1 with MscL-G22S-cGFP. (A) A plot of current against pressure from 7 cell-attached patches expressing both PIEZO1 channels and MscL-G22S-cGFP channels. **(B)** Normalized Boltzmann distributions from both channels from the currents shown in **(A)**. The $P_{1/2}$ ratio is 0.4 ± 0.06. This kind of approach using MscL as a standard has been used extensively for other MS channels. **(C)** Shows a raw record of activity induced by two negative pressure pulses of 350 ms in duration. The first saturates PIEZO1 channels in the patch the second also saturates PIEZO1 channels in the patch but in addition generates sufficient membrane stress to gate MscL-G22S-cGFP channels. (Data points represent mean ± S.E.M; n=6)
